# Molecular Properties and New Potentials of Plant Nepenthesins

**DOI:** 10.3390/plants9050570

**Published:** 2020-04-29

**Authors:** Zelalem Eshetu Bekalu, Giuseppe Dionisio, Henrik Brinch-Pedersen

**Affiliations:** Department of Agroecology, Research Center Flakkebjerg, Aarhus University, DK-4200 Slagelse, Denmark; giuseppe.dionisio@agro.au.dk (G.D.); hbp@agro.au.dk (H.B.-P.)

**Keywords:** Plants, aspartic proteases, nepenthesins, molecular properties, industrial applications, trait improvement

## Abstract

Nepenthesins are aspartic proteases (APs) categorized under the A1B subfamily. Due to nepenthesin-specific sequence features, the A1B subfamily is also named nepenthesin-type aspartic proteases (NEPs). Nepenthesins are mostly known from the pitcher fluid of the carnivorous plant *Nepenthes*, where they are availed for the hydrolyzation of insect protein required for the assimilation of insect nitrogen resources. However, nepenthesins are widely distributed within the plant kingdom and play significant roles in plant species other than *Nepenthes*. Although they have received limited attention when compared to other members of the subfamily, current data indicates that they have exceptional molecular and biochemical properties and new potentials as fungal-resistance genes. In the current review, we provide insights into the current knowledge on the molecular and biochemical properties of plant nepenthesins and highlights that future focus on them may have strong potentials for industrial applications and crop trait improvement.

## 1. Introduction

Aspartic proteases (APs, EC 3.4.23) comprise the second largest family of plant proteases. Plant APs have been identified from a wide range of plant species and tissues as extensively reviewed in Simões and Faro [1]. They are known to have important roles in storage protein processing [2,3], nitrogen remobilization [4,5,6], sexual reproduction [7,8,9], biotic and abiotic stress responses [10,11,12,13], and senescence and programmed cell death (PCD) [9,14,15]. In the MEROPS database (https://www.ebi.ac.uk/merops; [16]), APs are grouped into sixteen families, but the plant APs are distributed among A1, A3, A11, and A12 families of clan AA and family A22 of clan AD. The majority of plant APs identified so far belong to the A1 family. Based on their primary structure and distinct sequence features, the plant A1 AP family is subdivided into A1A (pepsin-type APs (PEPs)) and A1B (nepenthesin-type APs (NEPs)) subfamilies. The NEPs contain the only described extracellular proteases of plant origin, the nepenthesin.

Nepenthesin (EC 3.4.23.12), named after the carnivorous plant *Nepenthes*, represents a distinct group of proteases from the A1B subfamily. They are primarily reported from the pitcher fluid of *Nepenthes* plants [17,18] and account for the majority of the fluid protease activity [19]. Purification and characterization of proteases from the pitcher fluid has confirmed that the protease activity indeed is ascribed to nepenthesins [20,21,22]. Subsequently, cDNA clones of nepenthesins were characterized from *Nepenthes alata* [5] and *N*. *gracilis* [23]. Furthermore, nepenthesins were directly purified and characterized from the pitcher fluid of *N*. *distillatoria* [23] and other carnivorous plants [24]. 

Though similar aspartic proteases have also been isolated from the digestive fluid of carnivorous Caryophyllales (*Dionaea*, *Drosera capensis*) and *Cephalotus follicularis*, they were described as dionaeasin, droserasin, and cephalotusin [25,26]. However, since they share high sequence homology and biochemical property to nepenthesins, they can be named as nepenthesins [27,28]. Apart from carnivorous plants, nepenthesins have also been reported from *Arabidopsis thaliana* [7,29] and *Oryza sativa* [30]. In 2008, Takahashi and his colleagues studied the expression of nepenthesin genes in different *A*. *thaliana* tissues [29]. They found expression in various tissues, including leaves, stems, seeds and pods. This could indicate that nepenthesins involve in multiple physiological processes both in the vegetative and reproductive stages. In this regard, they could mainly be linked protein turnover from the old tissues to the newly growing tissues. 

In the current review, we summarize the current knowledge on the molecular structure, regulation and biochemistry of plant nepenthesins. In view of the recent findings, the review also describes the potentials of nepenthesins in both industrial applications and crop trait improvement. The information highlighted in this review will improve our understanding on the roles of nepenthesins in plant development and defense. 

## 2. Molecular Structure of Plant Nepenthesins

**Figure 1:**
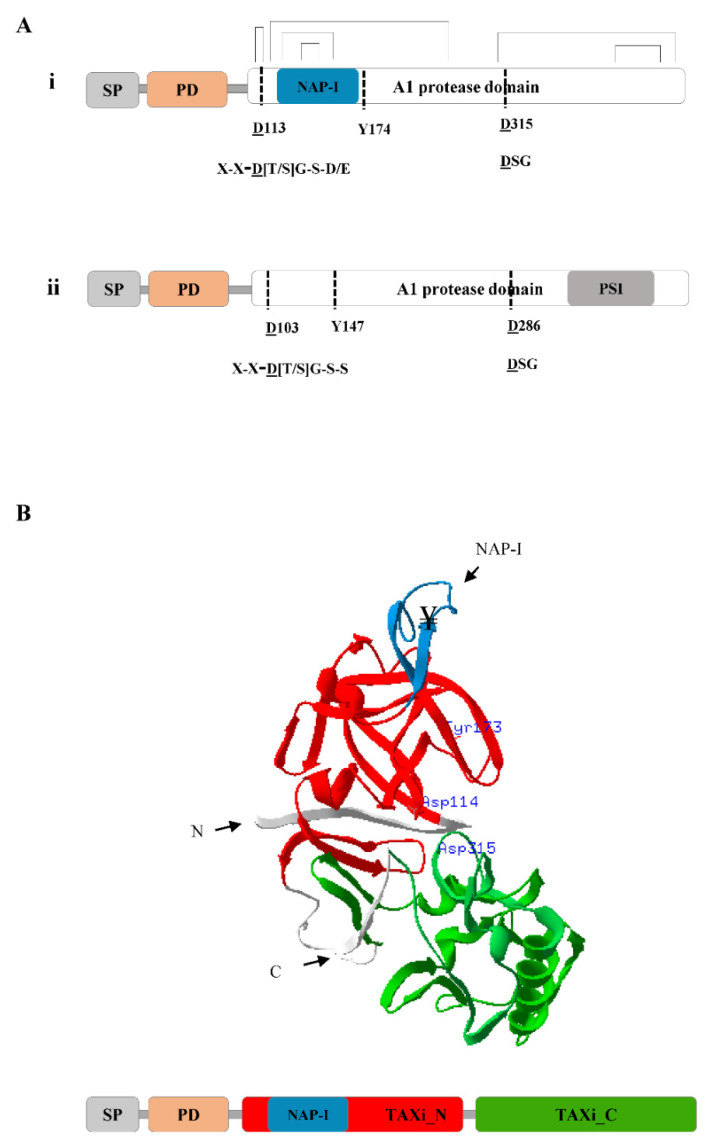
Schematic representation of the structure of nepenthesins (NEPs) and phytepsins (PEPs). (**A**) The primary structure organization of *N*. *gracilis* nepenthesin-2 (MER0031641) (i) and *Cynara cardunculus* phytepsin (MER0004937) (ii). Both proteins contain a signal peptide (SP), a prodomain (PD) and the A1 protease domain. The protease domain contains the two catalytic Asp residues (underlined) and the “flap” Tyr residue. The position of the PSI and NAP-I sequences are indicated for phytepsin and nepenthesin, respectively. The PEP and NEP signature motifs that contain the two catalytic Asp residues are indicated corresponding to the two Asp residues for both phytepsin and nepenthesin, (Ø- hydrophobic residue, D- Asp, T-Thr, G- Gly, Ser, E-Glu; [31]). (**B**) The tertiary structure of *N*. *gracilis* nepenthesin-2 protein predicted using Swiss PDB viewer [34]. The 3D annotation is based on the HMM based profile scan of the primary structure (shown below). In the primary structure, the position of the SP, PD, TAXi_N and TAXi_C, NAP-I sequence, and structurally important six disulfide bridges are shown. Two of the disulfide bridges are located in the NAP-I sequence. The TAXi_N and TAXi_C domains and the NAP-I sequence are colored in the 3D structure, consistent to the primary structure. The N- and C- termini and the active site residues (D113 Y174 D315) of the protein are also shown.

The primary structure of nepenthesins is comparable to the structure of pepsin, but few characteristic differences have been identified (Figure 1A). The precursor of nepenthesins contain an N-terminal signal sequence for secretion, a prodomain sequence of about 40 amino acids, two conserved catalytic Asp residues and a conserved Tyr residue that forms the so-called “flap” [1]. The two catalytic Asp residues are conserved in the Asp-Thr/Ser-Gly (D[T/S]G) and Asp-Ser-Gly (DSG) sequence motifs. The Tyr (Y) residue in the “flap” controls substrate specificity and binding. Nepenthesins are also characterized by the presence of a 20–30-residues-long NAP-I sequence (Figure 1A,i). This is different from the 100 residues long plant specific insertion sequence (PSI) that is specific to the A1A subfamily (Figure 1A,ii). Both the NAP-I and PSI are Cys-rich segments inserted at the N-and C-terminus of the protein, respectively. The NAP-I is usually placed between Trp39 and Tyr75 in the polypeptide (according to pepsin numbering) [23] and is often part of the mature enzyme [1,31]. 

NAP-I is characterized by its formation of two more disulfide bridges in the mature protein than the corresponding PEPs (Figure 1A,ii). The additional disulfide bridges in NAP-I contribute to the unusual stability of nepenthesins for a wider range of pH and temperature [32]. This gives nepenthesins the exceptional biochemical property to digest insect structures over long time without losing proteolytic activity. However, there are significant variations in the NAP-I of nepenthesins among species [32]. This may be associated to the specific roles of NAP-I in intracellular targeting or functional regulation of the enzyme in each species. The distinctive sequence features of nepenthesins are conserved among other members of the A1B subfamily, as recently reviewed in Soares etal. [31]. 

Prediction of the domain composition and tertiary structure of nepenthesins reveals extended molecular and structural features. For instance, according to the profile Hidden Markov Models (HMM) scan of the *N*. *gracilis* nepenthesin-2 [33], its A1 protease domain is mapped to the two xylanase inhibitor domains, TAXi_N (xylanase inhibitor N-terminal) and TAXi_C (xylanase inhibitor C-terminal) (Figure 1B). The tertiary structure prediction discloses that the two xylanase inhibitor domains form a lobe and thereby creates the catalytic pocket [34]. The active site is located in the cleft between the two lobes containing two catalytic Asp residues, one from each lobe (Figure 1B). The “flap” structure made up of a β-hairpin structure completes the active site and participates in substrate binding. The N- and C-termini of xylanase inhibitors are equally necessary for creating the catalytic pocket for cleaving xylanases [35,36,37]. As mentioned above, the NAP-I sequence does not appear to be directly involved in substrate binding or catalysis. It may be indirectly involved in maintaining the active site structure or participate in other cellular activities. 

## 3. Regulation of Plant Nepenthesins

Nepenthesins are mostly expressed as inactive zymogens [23,32]. The prodomain is covalently bound to the active site of the enzyme, and acts as auto-inhibitor. The prodomain is not often part of the mature enzyme and has to be processed during zymogen maturation [31]. In most cases, the inactive zymogens are activated in acidic environments after removal of the prodomain. Autocatalytic processing of the prodomain is sufficient to release the active enzyme, as the prodomain is no more bound to the active site. The prodomain probably serves as a molecular “switch” to keep the proteases inactive until they reach their final subcellular destination and prevent untimely activation and proteolysis. In addition to the complex pH dependent intramolecular and intermolecular steps in the maturation of nepenthesins, a different prodomain processing and enzyme activation mechanism has been described for other members of NEPs. For instance, the active form of AtCDR1 and OsCDR1 recombinant enzymes have been reported without the irreversible removal of the prodomain [11,38]. In addition, the activation of CND41 depends on the interaction of the leucine-rich N-terminal region and positive charged residues in the active site [39,40]. So far, no study has reported these types of enzyme activation mechanisms for nepenthesins. Likewise, an in silico analysis of the *A*. *thaliana* APs revealed that the majority of nepenthesins are predicted to be located in the secretory pathway [41]. Therefore, they are probably be either in the vacuoles or in the extracellular space. Future structural studies on the nepenthesins may provide in-depth molecular information about other modes of activation and subcellular locations. 

## 4. Biochemistry of Plant Nepenthesins

Nepenthesins are active and stable over a wider pH and temperature range than porcine pepsin A and other PEPs [23,32]. Moreover, in addition to the number of disulfide bridges, the stability of nepenthesins has largely been correlated to the level of N-glycosylation [42]. The majority of nepenthesins function optimally at acidic conditions, but few exceptions with basic pH optima have been reported (Table 1). As described for other members of APs, nepenthesins are inhibited by pepstatin and diazoacetyl-D,L-norleucine methyl ester (DAN) (Table 1). DAN only inhibited the activity of nepenthesins in the presence of cupric ions [23,32]. The effect of metal ions on the activity of inhibitors and chelating agents needs to be studied further for the nepenthesins. In contrast to members of PEPs, nepenthesins are more sensitive to various chemicals, reducing and denaturing agents such as TCEP (tris (2-carboxyethyl)phosphine), urea, and guanidine hydrochloride [43]. The denaturing agents considerably improved the activity of DTT that acts on the disulfide bridges of nepenthesins. TCEP alone or in combination with other denaturing agents significantly reduces the activity of nepenthesins [42]. However, the level of susceptibility to these chemical agents significantly varies between nepenthesins. For example, recombinant nepenthesin II from *N*. *gracilis* was more resistant to denaturing and reducing agents than nepenthesin I [44]. Its level of resistance to these denaturants and reducing agents was as good as pepsin.

The overall mechanism of peptide bond cleavage is the same for both NEPs and PEPs [45]. They use water molecules that act as a nucleophile and activates the catalysis via electrostatic interactions formed between the two catalytic Asp residues. The catalytic Asp residues stabilize the oxyanion hole of aspartic proteases. Although nepenthesins seem to have similar cleavage specificity to members of PEPs such as pepsin A and cathepsin D, additional cleavage sites have been identified [32,46]. In both studies, nepenthesin cleaved the B chain of oxidized insulin at Leu6-Cya7, Glu13-Ala14, Leu15-Tyr16 and Tyr16-Leu17 and Phe24-Phe25 sites to a significant level (Table 1). Moreover, nepenthesins cleave at Leu6-Cya7 (Cya for cysteic acid), which has never been described for members of PEPs [32]. Although Leu residue at the P1 position appears to be the ideal site for nepenthesins, high rate of cleavage by nepenthesin has been observed when Phe, Leu, Met, Lys, Arg, and Pro are present at the P1 position [47]. In addition to the B chain of oxidized insulin, nepenthesins have also shown strong affinity for a wide range of substrates such as casein, haemoglobin, myoglobin, and fungal phytase protein (Table 1). 

## 5. Biological Roles of Nepenthesins in Carnivorous Plants

### 5.1. Nepenthesins Contributing to Plant Nutrition

Nepenthesins have been regarded as the main players in hydrolyzing insect protein and availing the nitrogen for assimilation by the plant [24]. Due to significant proteolytic activity in the fluid, carnivorous plants can derive 10%–80% of their total nitrogen from insects [53]. The efficiency hydrolysis varies depending on the occurrence of proteolytic enzymes and the type of trap employed by the plant. In this regard, nepenthesins represent the major proteolytic activity in the pitcher fluid [50]. As a result, carnivorous plants thrive in extremely low nitrogen environments. The regulation of nepenthesin activity in the digestive fluid has been examined in response to chemical stimuli and live prey [54]. Ammonium, protein, and live prey strongly induced the transcription of nepenthesins. Although ammonium induced the highest transcription of nepenthesins, the total protease activity is higher in response to protein and live prey [54]. The result indicated that carnivorous plants monitor and digest insect prey with the aid of nepenthesins. 

### 5.2. Nepenthesins Play a Role in Plant Defense

Despite characterization of nepenthesins from various carnivorous plants, not a single attempt has been made to study their role in crop plants. Recently, we have identified and characterized the nepenthesin-1 gene (*HvNEP-1*) from barley [52]. The recombinant HvNEP-1 (rHvNEP-1) reduces the activity of fungal phytases, enzymes that dephosphorylate the phytic acid substrate [55]. The rHvNEP-1 also suppresses biomass accumulation and 15-acetyldeoxynivalenol (15ADON) mycotoxin production from different strains of *Fusarium graminearum* [52]. The study reports that rHvNEP-1 significantly represses the expression of trichothecene (*TRI*) biosynthesis and regulatory genes in *F. graminearum*. Among the four *TRI* genes (*TRI4*, *TRI5*, *TRI6*, and *TRI12*) examined, rHvNEP-1 significantly affects the expression of *TRI4*, *TRI5*, and *TRI6* genes. The repression of *TRI6* expression directly influences biomass accumulation and mycotoxin production, as it regulates the transcription of *TRI* biosynthesis genes and genes related to nutrient assimilation in *Fusarium* species [56]. Likewise, the suppression of *TRI4* and *TRI5* genes reduces the synthesis and accumulation of the trichothecene precursors early in the *TRI* biosynthesis pathway [57,58]. 

The reliability of *HvNEP-1* in plant defense has been further examined by overexpressing in the endosperm of barley grains and infection with the two DON-Chemotype *F. graminearum* and *F. culmorum* species. Surprisingly, the overexpression of *HvNEP-1* in the endosperm of barley grains significantly reduces the infection and disease progression of *Fusarium* head blight disease (FHB), under both *Fusarium* species. Furthermore, the analysis of deoxynivalenol (DON), nivalenol (NIV) and zearalenone mycotoxins indicates that *HvNEP-1* overexpression substantially suppress the accumulation of DON in the grains than in the untransformed controls [59]. The level of DON accumulation in the grains of some transgenic lines has been far below the optimal level considered for human consumption. 

## 6. Potentials of Plant Nepenthesins

### 6.1. Industrial Applications: Tool for Digestion in Hydrogen/Deuterium Exchange Mass Spectrometry

Hydrogen/deuterium exchange mass spectrometry (HDX-MS) has become an important method to study the structure of proteins and their complexes [60,61]. The technique requires proteases that can perform fast digestion at lower pH. For this application, pepsin is used more often, but it offers moderately low efficiency under the HDX-MS optimal conditions. Nepenthesins can be used as a substitute to pepsin in the HDX-MS application as they exhibit optimal biochemical properties and slightly wider cleavage specificities than pepsin [44,47,49]. It has been shown that nepenthesin is at least 1400-fold more efficient than pepsin under HDX-MS competent conditions (Table 2). In addition to similar cleavage sites as pepsin, nepenthesin also effectively cleaves C-terminal to Lys, Arg, His, and Pro residues under natural conditions. In general, nepenthesin-based digestion gives more sequence coverage and shorter average peptide length than pepsin [47,62]. Despite a number of cleavage sites common for nepenthesins, additional cleavage sites have been identified for some nepenthesins [44]. For instance, both nepenthesin I and II isolated from *N*. *gracilis* share similar biochemical properties, including fast digestion at low temperature and pH, and cleavage specificity. However, nepenthesin II specifically cuts the C-terminal to Trp. 

Nepenthesins are ideal proteases to study the structural transitions of intrinsically disordered proteins and their complexes (Table 2). This has been envisaged by using nepenthesin-enriched secretions to analyze protein complexes involved in the non-homologous end-joining DNA damage repair pathway [47]. The study demonstrated the molecular mechanisms behind the interaction of the BRCT domains of Ligase IV to the full length and truncated form of XRCC4. Based on the peptide cleavage results from XRCC4 digestion, the interaction between the disordered C-terminal tail of XRCC4 and remote regions of the BRCT domains is required for complex formation. This has been possible due to the cleavage of XRCC4 at Pro and charged residues, which is impossible using pepsin [47].

### 6.2. Treatment of Celiac Disease

Celiac disease is a chronic autoimmune disease of the small intestine caused by the ingestion of dietary gluten. The common grains like wheat, barley and rye are characterized by high content of gluten proteins. Upon consumption of gluten proteins, celiac patients often develop villous atrophy of the small intestine, crypt hyperplasia, and mucosal inflammation [63,64]. Most gastric proteases digest proteins into peptides as small as 30-40 amino acid residues, which are still resistant to further digestion by intestinal proteases [65]. The 33-mer peptide, LQLQPFPQPQLPYPQPQLPYPQPQLPYPQPQPF, has been identified as the chief initiator of the inflammatory response to gluten in celiac patients [66,67]. This toxic peptide from gluten proteins contains six repetitive T-cell epitopes that arouses a potent T-cell response in celiac patients. The peptide is highly resistant to digestion by gastric pepsin, even under prolonged incubation. Therefore, celiac patients consider the exclusion of gluten-containing diets as the only lifetime treatment for the disease. However, avoiding gluten proteins from our daily menu can be more expensive and unpleasant. 

Numerous strategies have been devised to alleviate the problem related to gluten-intolerant individuals. Some of the approaches include enzymatic hydrolysis of gluten proteins and the creation of low α-gliadins using RNAi and CRISPR/Cas9 transgenic technologies [68,69,70,71]. Enzyme-based detoxification of gluten proteins has been described as one of the most promising therapeutic approach, as there is still growing concern regarding the use of transgenic technologies [72]. In this regard, plant nepenthesins have displayed an impressive ability to degrade gluten into non-toxic peptides more efficiently than stomach enzymes like pepsin [62,63]. More importantly, they degrade the 33-mer peptide into non-toxic peptides [62,63]. It has also been demonstrated that nepenthesin showed efficient antigen processing at total substrate to enzyme ratios higher than 12,000:1 (Table 2). This has a direct implication for therapeutic use, as it requires low amount of nepenthesins for efficient detoxification of gluten proteins. On the contrary, a barley cysteine endopeptidase-B2 has been reported to hydrolyze glutens into small peptides, but it requires the prolyl endoprotease from *Sphingomonas capsulate* to produce non-toxic peptides [68,73]. Overall, nepenthesins provide better platforms for gluten intolerance therapy as (1) they are isolated from plants, (2) they are easy to isolate or produce, (3) they have very high substrate catalytic efficiency, (4) they hydrolyze gluten proteins into small and harmless peptides, and (5) they can be prepared as tablets or other formulations to use as food additives in gluten intolerance therapy [63].

### 6.3. Trait Improvement of Crop Plants

Interestingly, nepenthesin homologues are distributed in a wide range of crop species like wheat, barley, and maize [16]. Based on the available molecular and biochemical information, nepenthesins can aid the improvement of important traits like disease resistance. The recent discovery of *HvNEP-1* as a defense gene against the fungal disease FHB provides additional genetic resource for the improvement of disease resistance trait in crop plants [59]. In the study, the relative expression of *HvNEP-1* in the overexpression lines positively correlates to FHB resistance, both in the greenhouse and semi-field conditions. The increase in FHB resistance is also accompanied by the reduction of mycotoxin accumulation in the grains. 

*Fusarium* mycotoxins have been the center of global concern for decades, due to their significant health risks to human and animals [74]. Their level in agricultural crops has been regulated in most countries worldwide [75]. Despite the present various control methods, the use of non-chemical methods like resistance breeding has been the focus of researchers, as it minimizes the production costs and environmental pollution. In this regard, *HvNEP-1* can assist FHB resistance breeding in the common crops like wheat and maize. 

## 7. Conclusions and Future Perspectives

Despite their extensive occurrence in plants, research on nepenthesins lags behind compared to other members of the A1B subfamily. Previous studies were limited to the biochemical characterization of nepenthesins isolated exclusively from carnivorous plants. The studies have linked nepenthesins mainly to the degradation of insect structures and mobilization of insect-derived nitrogen for plant nutrition. In addition to their role in plant nutrition, the recently characterized barley nepenthesin *HvNEP-1* adds an additional potential of nepenthesins in plants as a defense protein. Furthermore, a study has also implicated nepenthesins in the treatment of bacterial infections in the gastrointestinal tract [63], which directs another research front for improving human health.

To gain a better understanding of the biological roles of nepenthesins, more studies are needed on the identification and characterization of various nepenthesin homologues from plants. In this respect, the investigation of their molecular mechanisms, subcellular locations, biological substrates, mechanism of regulation, and nepenthesin-associated receptors and transcription factors unlocks more mysteries related to their biological function. The role of the TAXi_N and TAXi_C domains of the nepenthesins in the inhibition of microbial xylanases requires further study. As described for the PSI domain of A1A APs [76], the role of the NAP-I in enzyme regulation and other species-specific function should also be investigated. In this regard, the ever-increasing computational and analytical tools in the area of genomics, transcriptomics and proteomics, and the evolution of new genome editing systems like CRSPR/Cas provide additional platforms for studying plant nepenthesins. The obtained information can eventually be used to improve enzymatic properties for industrial applications and for crop improvement programs. 

## Figures and Tables

**Table 1:** Biochemical properties of nepenthesins from various carnivorous and crop plants.

Source (Species)	Proteases	Molecular Weight (kDa)	Specific Activity	Substrates	Optimum		Inhibitors	Substrate Specificity		References
					pH	T (°C)		Substrates	Cleavage Specificity	
*Nepenthes* sp.	Nepenthesin	n.d.	0.55 U mL^−1^	Casein	2.8	40	n.d.	Peptides (8)	Leu-Asp, Ser-Asp, Thr-Asp, Ala-Ala, Tyr-Asp	[21]
*N. maxima* *N. rafflesiana* *N. ampullaria* *N. x dyeriana* *N. x mixta* *Drosera peltata*	Nepenthesin	n.d.	n.d.	Casein	3.0	40	n.d.	Peptides (6)	Asp-Glu, Asp-Ala, Ala-Ala, Lys-Arg	[22]
*Nepenthes* sp.	Nepenthesin	n.d.	n.d.	Casein	2.9	40	n.d.	n.d.	n.d.	[48]
*N. macfarlanei*	Nepenthesin I Nepenthesin II	59.00 21.00	n.d. n.d.	Bovine fibrin Bovine serum albumin Horse-heart cytochrome c	n.d. n.d. 2.2	37 37 37	Pepstatin	Horse-heart cytochrome c	Lys-Ala, Glu-Asp, Glu-Thr, Lys-Thr, Gly-Gly, Leu-Phe	[6]
*N. distillatoria*	Nepenthesin I Nepenthesin II	51.00 45.00	874 U mg^−1^ 809 U mg^−1^	Acid-denatured haemoglobin	2.6 2.6	55 45	Pepstatin, DAN	Oxidized insulin B chain	Phe-Phe, Glu-Ala, Leu-Cya, Leu-Tyr, Tyr-Leu	[23]
*N. alata*, *Cephalotus follicularis* *D. muscipula* *D. capensis*	Nepenthesin I Nepenthesin II	n.d. n.d.	n.d. n.d.	Haemoglobin Haemoglobin Oxidized insulin B chain	2.5 3.0 3.5	47–57 60 47	Pepstatin	Oxidized insulin B chain	Leu-Tyr, Phe-Phe, Glu-Ala, Ala-Leu, Tyr-Leu, Tyr-Thr, Lys-Ala, Gly-Phe,	[25]
*N. alata*	Nepenthesin I Nepenthesin IIa Nepenthesin IIb	n.d. n.d. n.d.	n.d. n.d. n.d.	Haemoglobin	n.d. n.d. n.d.	n.d. n.d. n.d.	n.d. n.d. n.d.	n.d. n.d. n.d.	n.d. n.d. n.d.	[43]
*N. gracilis*	Nepenthesin 1	43.73	n.d.	Haemoglobin Myoglobin	2.5 n.d.	50–60 n.d.	n.d.	n.d.	n.d.	[42,49]
*N. mirabilis*	Nepenthesin I Nepenthesin II	n.d. 45.00	n.d. n.d.	PFU-093 (FRET peptide substrate)	8.0 8.0	42 42	Pepstatin A	n.d.	n.d.	[50]
*N. alata*	Nepenthesin I Nepenthesin II	n.d. n.d.	n.d. n.d.	PFU-093 (FRET peptide substrate)	8.0 8.0	42 42	Pepstatin A	n.d.	n.d.	
*N. reinwardtiana* *N. distillatoria* *N. eymae* *N. wittei* *N. hookeriana* *N. boschiana* *N. maxima*	Nepenthesin I Nepenthesin II	n.d. n.d.	n.d. n.d.	PFU-093 (FRET peptide substrate)	8.0 8.0	42 42	Pepstatin A	n.d.	n.d.	
N. gracilis *	Nepenthesin I Nepenthesin II	n.d. 37.50	n.d. n.d.	Haemoglobin	2.5 2.5	50 55	n.d.	XRCC4, XLF, PNK, BRCT, myoglobin	Ser-Ilu/Pro/Thr//Phe, Glu-Glu, Leu-Tyr, Phe-Phe, Glu-Ala, Ala-Leu	[44]
*N. rafflesiana*	Nepenthesin-1 Nepenthesin-2 Nepenthesin-3 Nepenthesin-4 Nepenthesin-5	47.11 46.63 49.09 48.99 48.81	n.d. n.d. n.d. n.d. n.d.	None	n.d. n.d. n.d. n.d. n.d.	n.d. n.d. n.d. n.d. n.d.	n.d.	n.d.	n.d.	[51]
*H. vulgare*	Nepenthesin-1 (HvNEP-1)	48.90	n.d.	*Aspergillus ficuum* phytase	5.0	40	Pepstatin A	n.d.	n.d.	[52]

n.d.: not determined; kDa: kilo Dalton; T: temperature; FRET (fluorescent resonance energy transfer). For N. gracilis * nepenthesin I and II, 43 potential cleavage sites were identified but a small list of them is presented in the table. Two columns of substrates are indicated in the table. The first column of substrates was used for the determination of optimum condition for enzymatic activity, whereas substrates in the second column were used for enzyme-specific activity analysis. Peptide (6)/peptide (8) is to denote the number of specific peptide fragments used in the studies.

**Table 2:** Comparison of the proteolytic efficiency of nepenthesin to other proteases used for industrial applications.

Applications	Proteases	Purification	Substrates	Molar Ratio (Protease: Substrate)	References
HDX-MS	Porcine pepsin	Reagent grade (Sigma)	XRCC4, XLF, BRCT, PNK, myoglobin, cytochrome C	1:0.00192	[47]
	Nepenthesin II	Crude	XRCC4, XLF, BRCT, PNK, myoglobin, cytochrome C	1:2.63	
Celiac treatment	CysProt EP-B2	Recombinant (*E*. *coli*)	α2-gliadin	1:10	[68]
	Nepenthesin II	Crude	α2-gliadin	1:12000	[62]
